# Diagnosis and Long-term Management of Uromodulin Kidney Disease

**DOI:** 10.7759/cureus.4270

**Published:** 2019-03-19

**Authors:** Emily Wheeler, Suresh Thomas

**Affiliations:** 1 Internal Medicine, West Virginia University School of Medicine, Morgantown, USA

**Keywords:** uromodulin kidney disease, familial juvenile hyperuricemic nephropathy, autosomal dominant tubulointerstitial kidney disease, gout, renal failure, end stage renal disease, esrd (end stage renal disease)

## Abstract

Uromodulin kidney disease (UKD) is a subtype of autosomal dominant tubulointerstitial kidney disease (ADTKD), and is a rare cause of renal failure and gout in young people. Although it is inherited in an autosomal dominant fashion, the gene mutation exhibits variable expressivity so the phenotype varies dramatically among affected individuals. While it is rare, it is important for physicians in the primary care setting to be able to recognize the disorder, initiate proper workup, and refer patients to nephrology teams that are equipped to manage the long-term needs of these patients. Eventually, most will progress to renal failure with necessary renal dialysis or kidney transplant. Kidney transplant is curative as the new kidney does not have the defective tubule cell gene. The case series that follows highlights the variable presentations of the disorder among members of the same family and the necessary long-term follow-up that will often be handled by the primary care provider in conjunction with the specialist team.

## Introduction

Autosomal dominant tubulointerstitial kidney disease (ADTKD) is a group of three rare genetic diseases that share a common feature of slowly declining kidney function starting in adolescence or young adulthood and leading to end-stage renal disease (ESRD). Other features common to all three subtypes include: bland urine sediment with signs of chronic kidney disease (CKD) and the possibility of renal medullary cysts. The most common subtype is uromodulin kidney disease (UKD) caused by mutations in the uromodulin (UMOD) gene that encodes uromodulin (Tamm-Horsfall protein). Defective uromodulin cannot be exported and so accumulates inside renal tubule cells and leads to cell death. UKD is also characterized by hyperuricemia and early-onset gout, although these may not be seen in all patients with this genetic subtype. The second subtype is Mucin-1 kidney disease (MKD) caused by mutations in the MUC1 gene that causes renal failure without other specific findings. The rarest form is caused by renin (REN) mutations (ADTKD-REN) and is characterized by anemia in childhood, hypotension, mild hyperkalemia, and hyperuricemia [1].

## Case presentation

**Figure 1:**
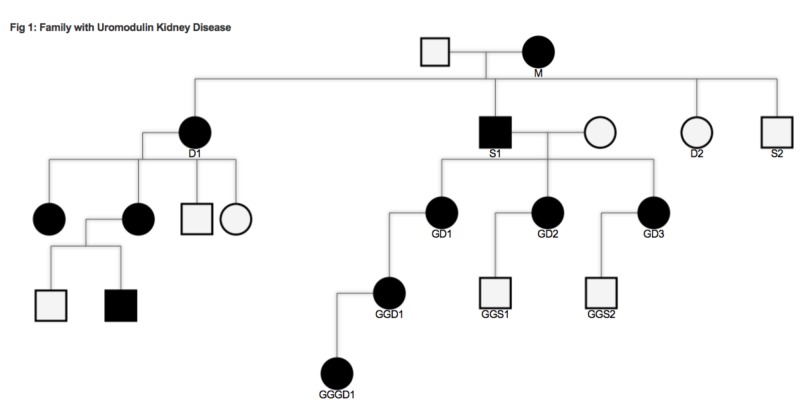
Family with uromodulin kidney disease M: Mother, S1: son, D1: daughter, GD1/2/3: granddaughter 1/2/3, GGD1: great-granddaughter, GGGD1: great-great-granddaughter

This report follows members of a family with documented UKD (Figure 1).

Patient S1 had gout and refractory hypertension for many years before being clinically diagnosed with a genetic kidney disorder in 1975. His mother (M), who is thought to have carried the UMOD mutation as well, had hypertension without gout and died at the age of 86 years. One of his sisters (D1) was also diagnosed with the disorder but little information about the clinical onset of disease in her family is available. In 1978, his three children (granddaughter (GD)1, 2, and 3) were tested and diagnosed using urine analysis and serum uric acid and creatinine levels. When it became available, genetic testing was done on two members of the family (GD2, great-granddaughter (GGD1)) and it found UMOD gene mutations. The table describes the clinical differences among the family members (Table 1).

**Table 1:** Variable presentations of uromodulin kidney disease in members of a family M: Mother, S1: son, D1: daughter, GD1/2/3: granddaughter 1/2/3, GGD1: great-granddaughter, GGGD1: great-great-granddaughter

Affected individual	Age at diagnosis	Age at institution of dialysis	Age at kidney transplant	Presence of gout	Hypertension	Age at death
M	Unknown	-	-	No	Yes	86
S1	47	42	47	Yes	Yes	54
GD1	17	42	44	Yes	Yes	-
GD2	16	45	46	Yes	Yes	-
GD3	8	33	36	Yes	Yes	-
GGD1	12	-	-	No	Yes	-
GGGD1	12	-	-	No	No	-

The severity of gout varies greatly from one member to another and some have hyperuricemia without gout. GGD1 is 49-years-old and has not yet required dialysis but her kidney function has declined to the point that she is eligible to be placed on the kidney transplant list. GD1, 2, and 3 all had successful kidney transplants which have effectively cured their hyperuricemia and gout. Since the disease is related to an intrinsic renal cell mutation, kidney transplant cures the disease without possible recurrence. They remain on blood pressure medications to protect the transplanted kidney.

## Discussion

While all three subtypes of ADTKD are rare, ~400 affected families have been identified in the United States. Early recognition of these patients ensures proper follow-up and management. Clinical suspicion for UKD should be raised in a young person with elevated serum creatinine, hyperuricemia or precocious gout, or hypertension. Due to the absence of hematuria or proteinuria, urinalysis is not helpful in diagnosis. As with any genetic disorder, taking a thorough family history is vital. De novo mutations resulting in UKD are very rare, so patients will usually have a strong family history of gout, chronic kidney disease, or kidney transplants. Patients should be referred for genetic testing to determine the mutations that are involved. Since disease severity, rate of progression, and end-point varies dramatically even among patients within the same family, management is determined on a case by case basis and involves symptomatic treatment. Patients with hyperuricemia or gout are started on allopurinol or febuxostat. Likewise, anemia, hyperphosphatemia, hypertension, and other manifestations of CKD are treated as they arise using the same guidelines as in patients without UKD. Unlike in proteinuric CKD, there has been no evidence that angiotensin converting enzyme inhibitors or angiotensin receptor blockers slow progression of CKD in patients with UKD. There are trials that show slowed progression to ESRD in kidney disease patients when they are started on allopurinol early in the disease course [2]. However, the results were not specific to UKD patients and there have not been adequate trials in this population. While there is variability in age of onset, UKD will almost invariably progress to ESRD. Preemptive renal transplantation in adults is considered when the glomerular filtration rate (GFR) is <20 mL/min/1.73 m2. A renal transplant is considered curative. Dialysis will need to be considered if donor kidney is not available and GFR drops enough that signs and symptoms of kidney failure become apparent (usually Stage 5 CKD) [3].

## Conclusions

Ultimately, genetic diagnosis and eventual renal transplant will happen at an established medical center capable of providing these services, but long-term management will often fall to the primary care physician. Optimal control of hypertension, hyperuricemia, and kidney function will determine a patient’s quality of life and future development of comorbidities secondary to their diagnosis.
